# Misdiagnosis of Psychosis and Obsessive-Compulsive Disorder in a Young Patient with Autism Spectrum Disorder

**DOI:** 10.1155/2023/7705913

**Published:** 2023-02-14

**Authors:** Jiangbo Ying, Melvyn Weibin Zhang, Sreedharan Geetha Sajith, Giles Ming-Yee Tan, Ker-Chiah Wei

**Affiliations:** ^1^Adult Neurodevelopmental Service, Department of Developmental Psychiatry, Institute of Mental Health, Singapore; ^2^Central Region, Institute of Mental Health, Singapore

## Abstract

Autism spectrum disorder (ASD) is a neurodevelopmental disorder characterized by deficits in social interaction and the presence of restricted and repetitive patterns of behavior. Making a first diagnosis of ASD in adults has certain difficulties, including inaccurate recall of developmental history and overlapping behaviors with other psychiatric conditions. This case study presents a young man who was assessed to have no major mental illness during his first visit to emergency services in a psychiatric hospital. During his second visit, he was initially assessed to have first episode psychosis, due to his possible delusional beliefs related to the insurance payout, social withdrawal, and strange behaviors, and then later he was assessed to have obsessive-compulsive disorder (OCD) instead of psychosis, because of his recurrent and intrusive thoughts. Eventually, his diagnosis was revised to ASD during outpatient follow-up after more comprehensive assessment. It is not easy to differentiate ASD from psychosis among some adult patients, even for expert psychiatrists. Cognitive rigidity in ASD may be similar to delusions in psychosis. Unusual behaviors in ASD can be confused with disorganized behaviors in psychosis. Differentiating ASD from OCD can be a complicated task as well, due to similarities between ASD and OCD. Restricted interests and repetitive behaviors in ASD may be perceived as obsessions and compulsions in OCD. Overall, diagnosis of ASD in adults requires comprehensive evaluation. Distinguishing symptoms of OCD and psychosis from autistic traits is critical for accurate diagnosis and optimal treatment. Although research in adult ASD has expanded alongside increased prevalence statistics over the past few years, more efforts to enhance the diagnostic processes in adult ASD are needed to reduce the challenges in this field.

## 1. Introduction

Autism spectrum disorder (ASD) is a neurodevelopmental disorder characterized by deficits in social interaction and the presence of restricted and repetitive patterns of behavior, with an onset in the early developmental period [1]. The prevalence of ASD is estimated to be 0.76%–1.68% [2]. Children and youth with ASD have service needs in various areas, including education, health, and family support [3]. Individuals with ASD can even have maladaptive behaviors, such as physical aggression, irritability, inattention, and poor sleep [4, 5]. It is recommended to perform standardized screening for ASD at 18 and 24 months of age with ongoing developmental surveillance [3]. The diagnosis of ASD requires clinical assessment, and its early identification and interventions are important in improving clinical outcomes [6, 7]. However, sometimes the diagnosis of ASD can be challenging, especially among adult individuals. Making a first diagnosis of ASD in adults has several difficulties, including inaccurate recall of developmental history, learnt coping strategies to conceal social communication deficits, and overlapping behavior with other psychiatric conditions [8].

There are similarities between the deficit symptoms of psychosis and symptoms of ASD [9], and it has been previously proposed that ASD and psychosis are on two ends of a spectrum related to the social brain [10]. Psychosis is a broad term to describe symptoms to a change in perception of reality [11]. Generally, these five domains need to be evaluated to diagnose patients with psychosis: delusions, hallucinations, disorganized speech, grossly disorganized or catatonic behavior, and negative symptoms [1]. Sometimes it is not easy to distinguish between psychosis and autistic traits. For example, it may be hard to differentiate between childish fantasies in ASD and delusional beliefs in psychosis [12]. Persons with ASD are also reported to experience increased levels of anomalous perception due to their sensory issues [13]. These experiences can be similar to hallucinations in psychosis.

There is also some overlap between obsessive-compulsive disorder (OCD) and ASD. OCD is a mental illness characterized by the presence of obsessions and/or compulsions, which are time-consuming or cause clinically significant distress or functional impairment [1]. It has been reported that ASD traits are more common in persons with OCD than in healthy population and are associated with poor functioning in OCD [14]. Obsessions in OCD are sometimes similar as restricted interests in ASD, while compulsions in OCD are often likened to repetitive behaviors in ASD [15]. The age onset of OCD is bimodal which peaks in childhood and again in adulthood, with an early age onset indicating more severe symptoms [16]. Thus, if an individual develops obsessive or compulsive symptoms in childhood, it may be difficult to differentiate it from restricted and repetitive behaviors in ASD.

We reported a young patient of ASD who was initially misdiagnosed with psychosis and OCD, and aim to highlight the diagnostic challenges in ASD. This case report adds to the growing literature of challenges in diagnosing ASD in adulthood.

## 2. Case Presentation

A 17-year-old junior college student was first brought to our hospital emergency services by his parents for anger management issues. He stayed with his parents, 9-year-old brother, and a domestic helper. Recently, he had quarreled with his brother and thrown his brother's bag down the rubbish chute. He was upset that there was a cockroach at home and demanded that his domestic helper must clean the entire house at night. This incident was so traumatic to the domestic helper that she resigned thereafter. He was described by his parents to be stubborn and spoiled since childhood. However, after further assessment at our emergency services, he was assessed to have no depressive or psychotic symptoms. There was no evidence of any major mental illness. He was not given any medication or follow-up appointment.

One year later, when the patient was 18 years old, he was brought by his parents to our hospital again. Over the past 3 months, he kept asking his mother to buy insurance and then commit suicide, so that the rest of the family could get the insurance payout of millions of dollars. He stayed in his room, searching online almost all day about insurance. He was also unkempt and kept buckets filled with his own urine in his room. He denied low mood or any unusual experiences. His parents shared that he had recently dropped out of the University. According to his parents, the patient's grandfather had a history of schizophrenia, but there was no family history of other mental illness, such as ASD or OCD. In view of his possible delusional beliefs related to the insurance payout, strange behavior of keeping buckets of urine, social withdrawal, functional decline, and family history of psychosis, the patient was then assessed to have first episode psychosis based on the criteria of the Diagnostic and Statistical Manual of Mental Disorders, Fifth Edition (DSM-5). He was admitted to the ward for further management, and he was given risperidone 1 mg every night.

During this admission, he reported that he had recurrent thoughts about insurance payout and described those thoughts were obsessive and wanted to stop the thoughts. He did not have any hallucinations. His diagnosis was then revised to OCD, and he was given fluvoxamine 50 mg every night. He was noted to have poor eye contact and robotic nature of speech during the admission, but he could have back-and-forth conversations and was able to interact well with other patients on the ward. His preoccupation about insurance payout improved on the ward and he was eventually discharged to follow-up in our clinic.

During the outpatient follow-up, possibility of ASD was suspected in view of his robotic speech and persistent preoccupations about specific topics. He was eventually referred to our Adult Neurodevelopmental Service (ANDS) for more comprehensive assessment. The assessment performed at the ANDS clinic was guided by the Royal College of Psychiatrists Diagnostic Interview Guide for Assessment of Adults with Autism Spectrum Disorder. The detailed history from the patient and his parents revealed that he had poor social skills. He would attend birthday parties and play with others, but did not develop any meaningful friendships. Once he attended a prewedding ceremony of his relatives and he asked the bride and groom why they were going through the wedding ceremony, instead they could just sign a document to save all the expenses. He would also miss verbal or nonverbal cues. For example, when his father raised his voices, it would have little impact on him, as he would not understand that his father was angry. He had been stubborn since childhood and would get preoccupied with certain topics. He did not like sudden changes in plan. His parents shared that he had delay in speech development and he received speech therapy during childhood. His school records in primary school suggested that he needed to take great effort in team work and communication skills. To further aid the diagnostic process, he also completed the autism spectrum quotient (AQ) and empathy quotient (EQ). His AQ score was 17 which was not indicative of ASD, and his EQ score was 17 which showed poor empathy skills. Overall, his history and presentation indicated significant impairments in social interaction and communication starting from early childhood. He also had significant rigidity in thinking and often got preoccupied with certain themes. These findings were consistent with the diagnosis of ASD. As his preoccupations and ruminations appeared to be part of ASD, an additional diagnosis of OCD was not warranted.

## 3. Discussion

ASD is often difficult to diagnose unless there is a comprehensive clinical assessment with early childhood and developmental history. In the case presented here, the patient was assessed to have no major mental illness during his first visit to our emergency services. During his second visit, he was initially assessed to have first episode psychosis, due to his possible delusional beliefs related to the insurance payout, social withdrawal, and strange behaviors, and then later he was assessed to have OCD instead of psychosis, because of his recurrent and intrusive thoughts. Eventually when he was an adult, his diagnosis was revised to ASD after more comprehensive assessment.

It is not easy to differentiate ASD from psychosis among some patients, even for expert psychiatrists [17]. Lack of emotional reciprocity in ASD is frequently seen in patients with negative symptoms of psychosis. Cognitive rigidity in ASD may be similar to delusions in psychosis. Some unusual behaviors in ASD can be confused with disorganized behaviors in psychosis. The overlap between ASD and psychosis has been recognized since the time of Kanner [18]. However, there are still some factors to help in differentiating these two conditions. While the fixed beliefs in ASD may result from the difficulties understanding the rules of social interactions, the delusions in psychosis may occur based on the misperception of others' intention [19]. Lack of emotional reciprocity in ASD can be defined as the absence of mutual and symmetrical exchange between individuals while interacting with each other [20], while the blunted effect in negative symptoms of psychosis is understood as a decrease in the expression of emotion and reactivity to events [21]. Furthermore, ASD is an early onset and lifelong neurodevelopmental disorder [22], while psychosis, such as schizophrenia, typically appears in early adulthood, usually with a prodromal period preceding the first episode [23].

Differentiating ASD from OCD can be a complicated task as well. There are similarities between ASD and OCD. One study assessed a sample of 36 participants with a third having OCD, a third having ASD and OCD, and a third having OCD with Tourette's syndrome, and found no significant differences in obsessive compulsive symptoms [24]. On one hand, up to 86% of persons with ASD can have compulsive behaviors, such as repetitive tapping and hand washing [25]. On the other hand, about 20% of the individuals with OCD had pronounced ASD traits [26]. It has also been reported that there are similar levels of sameness behavior and repetitive movements in individuals with ASD and individuals with OCD [27]. However, there are several considerations which can facilitate the separation of ASD and OCD. While persons with OCD may feel their obsessions are distressing, persons with ASD may not believe that their intrusive thoughts can cause harm [28]. It has also been reported that ASD may present lower frequencies of contamination and aggressive obsessive thoughts and checking behaviors compared to OCD [29]. Moreover, autistic repetitive behaviors may be perceived as pleasurable experiences, while compulsions in OCD are often egodystonic and are resisted by the patients [30]. Additionally, autistic behaviors may be sensory seeking, but symptoms in OCD are usually driven by anxiety [31].

Currently there are no clear ASD biomarkers or diagnostic measures, and ASD is diagnosed based on fulfilment of descriptive criteria. Some screening tools may help to identify individual with ASD early, such as social communication questionnaire (SCQ), social responsiveness scale (SRS), and autism spectrum screening questionnaire (ASSQ) [32–34]. In our case report, the AQ and EQ questionnaires are used to help ASD diagnosis. In clinical practice, the AQ is a widely used tool to quantify autistic symptoms [35]. The AQ is designed as a self-report measure for ASD symptoms [36–38]. The AQ has 50 items with a maximum score of 50 and a cut off score of 26 [39]. The EQ is used as a self-report screen tool to assess impairments in empathy, with the majority of autistic persons scoring lower than 30 on the scale [40]. Other than questionnaires, there are several clinical practice guidelines for diagnosis of ASD. The Royal College of Psychiatrists Diagnostic Interview Guide for Assessment of Adults with Autism Spectrum Disorder is one of the useful tools for diagnosis of ASD in adults [41]. This guide is designed to help clinicians to conduct a comprehensive diagnostic interview to ascertain whether an adult has ASD.

In conclusion, diagnosis of ASD in adults requires comprehensive evaluation. Distinguishing symptoms of OCD and psychosis from autistic traits is critical for accurate diagnosis and optimal treatment. Although research in adult ASD has expanded alongside increased prevalence statistics over the past few years, more efforts to enhance the diagnostic processes are needed to reduce the challenges in this field.

## Data Availability

The data used to support the findings of this study are included within the article.
